# Transcriptomic and metabolomic analyses to study the key role by which *Ralstonia insidiosa* induces *Listeria monocytogenes* to form suspended aggregates

**DOI:** 10.3389/fmicb.2023.1260909

**Published:** 2023-10-12

**Authors:** Xifeng Zuo, Meilin Chen, Xinshuai Zhang, Ailing Guo, Si Cheng, Rong Zhang

**Affiliations:** ^1^National Research and Development Center for Egg Processing, College of Food Science and Technology, Huazhong Agricultural University, Wuhan, Hubei, China; ^2^Liunan District Modern Agricultural Industry Service Center of Liuzhou City, Liuzhou, Guangxi, China

**Keywords:** *Ralstonia insidiosa*, *Listeria monocytogenes*, suspended aggregates, transcriptome, metabolome, sterile supernatants

## Abstract

*Ralstonia insidiosa* can survive in a wide range of aqueous environments, including food processing areas, and is harmful to humans. It can induce *Listeria monocytogenes* to form suspended aggregates, resulting from the co-aggregation of two bacteria, which allows for more persistent survival and increases the risk of *L. monocytogenes* contamination. In our study, different groups of aggregates were analyzed and compared using Illumina RNA sequencing technology. These included *R. insidiosa* under normal and barren nutrient conditions and in the presence or absence of *L. monocytogenes* as a way to screen for differentially expressed genes (DEGs) in the process of aggregate formation. In addition, sterile supernatants of *R. insidiosa* were analyzed under different nutrient conditions using metabolomics to investigate the effect of nutrient-poor conditions on metabolite production by *R. insidiosa*. We also undertook a combined analysis of transcriptome and metabolome data to further investigate the induction effect of *R. insidiosa* on *L. monocytogenes* in a barren environment. The results of the functional annotation analysis on the surface of DEGs and qPCR showed that under nutrient-poor conditions, the *acdx, puuE*, and *acs* genes of *R. insidiosa* were significantly upregulated in biosynthetic processes such as carbon metabolism, metabolic pathways, and biosynthesis of secondary metabolites, with Log_2_FC reaching 4.39, 3.96, and 3.95 respectively. In contrast, the Log_2_FC of *cydA, cyoB*, and *rpsJ* in oxidative phosphorylation and ribosomal pathways reached 3.74, 3.87, and 4.25, respectively. Thirty-one key components were identified while screening for differential metabolites, which mainly included amino acids and their metabolites, enriched to the pathways of biosynthesis of amino acids, phenylalanine metabolism, and methionine metabolism. Of these, aminomalonic acid and Proximicin B were the special components of *R. insidiosa* that were metabolized under nutrient-poor conditions.

## 1. Introduction

*Ralstonia insidiosa* is a non-fermenting, aerobic, Gram-negative bacterium that often causes infections as a conditional pathogen (Coenye et al., 2003; Alaşehir et al., 2020). As an environmental bacterium, *R. insidiosa* can be found in a wide range of aqueous environments such as industrial water, laboratory pure water, and hospital water systems, as well as in rivers, ponds, and soils. It is equally well suited to survive in low-nutrient surroundings (Anversa et al., 2022). Current studies show that *R. insidiosa* isolated from the fresh fruit processing environment plays a bridging role in the biofilm formation of many foodborne pathogens such as *Salmonella enterica, Listeria monocytogenes*, and *Escherichia coli* (Liu et al., 2015, 2016). Seemingly harmless *R. insidiosa* has been isolated from patients with cystic fibrosis rings in the lungs and patients with bone surgeries. It was also found in immunocompromised patients who were on hemodialysis due to sepsis in medicine (Ryan et al., 2011; Fang et al., 2019). *R. insidiosa* has gradually gained attention due to its specific ability to cause infections and facilitate biofilm formation in other bacterial strains (Ryan and Adley, 2014).

*Listeria monocytogenes* is a zoonotic foodborne pathogen that is widely distributed on soil surfaces and water environments (Kallipolitis et al., 2020). Studies have shown that *L. monocytogenes* in a biofilm state is significantly more recalcitrant to several physicochemical stresses and can evade antibiotics and the body's immune system defenses (Cabo et al., 2022). As a result, it can cause continuous contamination and lead to serious food safety incidents (Halbedel et al., 2018).

Microorganisms rarely exist alone in any type of ecosystem. When multiple microorganisms coexist at interfaces such as solid-liquid or gas-liquid, they often aggregate through the mediation of their own secreted extracellular polymeric substances (EPS) and the interfacial interactions (Hall-Stoodley et al., 2004). There are three states of such aggregation, which include adhering to solid surfaces to form biofilms, forming films on gas-liquid surfaces, and forming suspended aggregates inside liquids (Leff et al., 2016; Dong et al., 2020). Among them, suspended aggregates are a common way of life for bacteria (Cai et al., 2019). In the past, suspended aggregates were considered as another form of biofilm, where microbial cells strongly expressed EPS components (White et al., 2008; Ramalingam et al., 2013). However, current studies have found that the biomass and ultrastructural performance of suspended aggregates and biofilms formed by the same strains are different (Chen et al., 2021), implying that the formation of suspended aggregates is regulated by different mechanisms (Secor et al., 2018). In such well-organized structures, microorganisms are better adept at resisting environmental stresses, such as disinfectants and biocides (Chylkova et al., 2017). The microbial cells in the aggregates secrete strong EPS that attract and wrap the bacteria and interact to form a granular substance visible to the naked eye (Grantcharova et al., 2010; Klancnik et al., 2020). The aggregation between the bacterial cells depends on the environment and on the different bacterial species (Trunk et al., 2018; Nwoko and Okeke, 2021). With the help of other bacteria, some bacteria can aggregate to form flocs (Simões et al., 2007). For example, *L. monocytogenes* cells, which cannot form suspended aggregates on their own, can be induced by *R. insidiosa* (Guo et al., 2016). Meanwhile, the production of suspended aggregates is currently reported to be the underlying cause of chronic diseases, suggesting the need to focus on the causes and processes that generate aggregates in the coexistence of multiple microorganisms in the environment.

Based on preliminary research (Guo et al., 2016; Chen et al., 2021), we further analyzed the formation of aggregates at different times under different conditions and observed their ultrastructure. Transcriptome sequencing was used to analyze the transcript changes in *R. insidiosa*, study its aggregates and how it induces *L. monocytogenes* to form aggregates, and finally screen key genes and metabolic pathways from a molecular perspective. The induction effect of *R. insidiosa* on *L. monocytogenes* not only depends on the direct contact of the bacteria but also on the secondary metabolites produced by *R. insidiosa* during the growth process that can induce *L. monocytogenes* to form aggregates. Therefore, we used metabolomics to analyze the secondary metabolites produced by *R. insidiosa* to screen the key metabolites and metabolic pathways from a material perspective. The combined analysis of dual-omics can more effectively identify common enrichment pathways at the metabolic and gene levels and screen candidate genes based on the results of the metabolite-gene correlation. It reveals the underlying mechanism and relationship of how environmental bacteria *R. insidiosa* promotes the formation of aggregates of *L. monocytogenes*. It also provides a reference for bacterial aggregation and its transition between different ecological niches to form multicellular communities.

## 2. Materials and methods

### 2.1. Bacterial strains and activation procedure

*Ralstonia insidiosa* ATCC 49129 was purchased from the American Type Culture Collection; *Listeria monocytogenes_*100, where “100” is the name of the designated strain, was isolated from milk samples in our laboratory. It is a serotype 1/2a strain with strong biofilm and suspended aggregate formation ability. Prior to the experiments, strains stored at −80°C in a tryptic soy broth (TSB; Hope Bio-Technology, Qingdao, China) containing 40% glycerol were activated and separated on tryptic soy agar plates (TSA; Hope Bio-Technology, Qingdao, China) for 24 h at 37°C. The single colony was transferred into 20 mL TSB overnight at 37°C, and the concentration of each final bacterial suspension was adjusted to 10^8^ CFU/mL.

### 2.2. Formation of suspended aggregates

#### 2.2.1. Suspended aggregate formation by *R. insidiosa*

Two hundred microliters of *R. insidiosa* or *L. monocytogenes* (10^8^ CFU/mL) and 100 μL of *R. insidiosa* with 100 μL of *L. monocytogenes* bacterial suspensions were separately cultured and co-cultured into the plastic Petri dishes (with a diameter of 90 mm), containing 20 mL of either TSB or 10% TSB (diluted 10 times) medium. Thereafter, the Petri dishes were incubated at 28°C for 24 h under agitation (40 r/min) and were checked every 6 h for the formation of suspended aggregates.

#### 2.2.2. Suspended aggregates formation of *L. monocytogenes* induced by RIS

The single colony of *R. insidiosa* was inoculated in 20 mL TSB and 10% TSB for 24 h at 28°C and 160 r/min. It was centrifuged at 6,000 r/min for 20 min, and the cell supernatant was filtered through a 0.2 μm aPES membrane (ThermoFisher, USA). The filtered sterile supernatant of *R. insidiosa* (RIS) was streaked on a TSA medium to ensure sterilization. After that, 10 mL RIS (100% or 10%) was mixed 1:1 with the corresponding concentrations of 10 mL TSBs (100% or 10%) containing cultures with 200 μL of *L. monocytogenes*. The Petri dish was incubated at 28°C for 24 h under agitation (40 r/min) and was checked every 6 h.

#### 2.2.3. Measurement of aggregates index

The aggregates (with a diameter >0.1 cm) were collected in sterilized centrifuge tubes using a trimmed pipette tip. We then gently shook the remaining broth in the Petri dishes and measured the OD_600_; the absorbance values were defined as OD_s_. The remaining broth in the Petri dish was pipetted back into the corresponding centrifuge tube containing the suspended aggregates and then vortexed to disperse and mix thoroughly; the measured absorbance values were defined as OD_t_, and we carried out three tests for each group. The aggregation index of the suspended aggregates was calculated according to the following equation (Li et al., 2021), which indicated the proportion of bacteria (among all bacteria) that tended to aggregate in a Petri dish (planktonic bacteria and aggregates):

$$\begin{array}{l} Aggregation\;index\;=\left[\frac{ODt-ODs}{ODt}\right]\times 100\% \end{array}$$

### 2.3. Microscopic observation of suspended aggregates

Following the methods described in 2.2.1 and 2.2.2, the suspended aggregates formed after 24 h were collected, and the planktonic bacteria were removed by rinsing with sterile physiological saline solution three times. After 5 min of centrifugation at 4,000r/min, the aggregates were fixed with 2.5% (vol/vol) glutaraldehyde (pH 7.4, 0.02 M) and placed in a refrigerator at 4°C for a night. Half of the immobilized aggregates were dehydrated with gradient ethanol solution (50%, 70%, 90%, 95%, 100%; vol/vol) for 10 min each time. The dehydrated aggregates were air-dried at room temperature to fully evaporate the organic reagents and then freeze-dried for 1 d. The lyophilized samples were gold sprayed and then scanned using a biological scanning electron microscope (SEM; JSM-6390LV, JEOL, Tokyo, Japan). The remaining half of the samples were infiltrated with low-viscosity embedding resin (Spurr, 1969) and ethanol and polymerized at 60°C for 48 h. Sections of 90 nm in thickness were cut on a Reichert Ultracut microtome with a diamond knife (Diatome) and stained with 4% aqueous uranyl acetate and 3% aqueous lead citrate. Finally, these were observed using transmission electron microscopy (TEM; H-7650, Hitachi, Tokyo, Japan).

### 2.4. Transcriptomic analyses of aggregates formation of *R. insidiosa*

#### 2.4.1. Preparation of sequencing samples

According to the method described in 2.2.1, *R. insidiosa* was cultured separately in 20 mL of either TSB or 10% TSB (diluted 10 times) medium and co-cultured with *L. monocytogenes*. We collected the suspended aggregates and washed the surrounding planktonic bacteria three times with saline. The *R. insidiosa* sample in TSB was numbered X, and that in 10% TSB was numbered Y. Meanwhile, the *R. insidiosa* co-cultured with *L. monocytogenes* was numbered Z, and each group of samples was repeated three times.

#### 2.4.2. Extraction of RNA and library construction

RNA was extracted using TRIzol reagent (Invitrogen, Carlsbad, CA, USA) based on the method described by Zhang et al. (2020). The concentration and OD_260/280_ of total RNA were tested using an N60Touch ultra-micro spectrophotometer (Implen, Germany). All RNAs used for library preparation were detected by the Agilent 2100 method and determined to have an RNA integrity number (RIN) of 6.5 and above. The construction of the RNA-seq library was mainly based on the manufacturer's manual and Srinivasan et al. (2020). Libraries with different indexes were multiplexed and loaded on Illumina HiSeq equipment following the manufacturer's manual (Illumina, San Diego, CA, USA). Bowtie2 (v2.2.6) was utilized to index the reference genome sequence of *R. insidiosa* (https://www.ncbi.nlm.nih.gov/nuccore/NZ_VZPV00000000.1). All transcriptome raw data were stored at the National Center for Biotechnology Information (NCBI) and Sequence Read Archive (SRA) database, registered as PRJNA996090.

#### 2.4.3. Transcriptome data analysis

Gene expression was calculated using Htseq software (v0.6.1p1), which uses FPKM (expected number of fragments per kilobase of a transcript sequence per million of base pairs sequenced) to calculate gene expression (Liu et al., 2020). For samples with biological duplication, differentially expressed genes (DEGs) analysis was performed using DEseq2 (v1.6.3) from the Bioconductor software package. Based on Benjamini's method (Benjamini and Hochberg, 2000), the results were screened according to the differential significance criteria—Log_2_Foldchange (Log_2_FC) ≥ 2, p-value (padj) ≤ 0.05, and the upregulation of the DEGs was counted. GO and KEGG functional classification and enrichment analysis were done for DEGs, and the screening criterion was a false discovery rate (FDR) ≤ 0.01 (Biswas and Chattopadhyay, 2022).

### 2.5. Metabolomic analysis of *R. insidiosa* sterile supernatant

#### 2.5.1. Preparation of samples

A single colony of *R. insidiosa* was inoculated in 20 mL of 3 concentrations of TSB medium (100%, 20%, or 10%) and were numbered B, C, and D, respectively, at 28°C for 24 h in a shaker at 160 r/min. After being centrifuged at 6,000 r/min for 20 min, the supernatant was filtered through a 0.2 μm filter, and the sterile supernatant of *R. insidiosa* (RIS) was streaked on a TSA medium to ensure sterilization. Two milliliters of RIS and sterile TSB were taken, snap frozen in liquid nitrogen, and stored at −80°C, where TSB was used as control and numbered as A. The samples were replicated three times.

#### 2.5.2. Extraction of metabolites and UHPLC/MS/MS analysis

The samples were removed from the −80°C freezer and were placed on ice to thaw; all subsequent operations were required to be performed on ice. After thawing, the samples were vortexed for 10 s to mix well, and 50 μL of the samples were transferred into the corresponding numbered centrifuge tubes. One hundred and fifty microliters of methanolic extract of 20% acetonitrile was added and vortexed for 3 min and then centrifuged at 12,000 r/min for 10 min at 4°C. After that, 150 μL of the supernatant was pipetted into a correspondingly numbered centrifuge tube and allowed to stand for 30 min at −20°C in a freezer. The supernatant was centrifuged at 12,000 r/min for 3 min at 4°C, and 120 μL of the supernatant was pipetted into the liner tube of the corresponding injection vial and used for further analysis.

The data acquisition instrumentation system mainly consisted of ultra-high-performance liquid chromatography and Tandem mass spectrometry (QTRAP^®^, https://sciex.com/). LIT and triple quadrupole (QQQ) scans were obtained on a triple quadrupole linear ion trap mass spectrometer (QTRAP) and an AB4500 QTRAP UPLC/MS/MS system (Yu et al., 2021; Klomkliew et al., 2022).

All sample extracts were subjected to LC-QTOF-MS/MS experiments to analyze broadly targeted metabolites. Accurate characterization and extraction of multiple ion pair information and the retention time of the identified metabolites were based on the self-built targeting specimen database MWDB (with the secondary spectrum, retention time), the MHK database (containing Metlin, HMDB, KEGG database, secondary spectrum, retention time), and MetDNA. Along with Metware's target database, triple quadrupole mass spectrometry and Q-Trap were used for accurate quantification of population samples.

#### 2.5.3. Data evaluation

Principal component analysis (PCA) was used to analyze the data of the detected metabolites to obtain a preliminary understanding of the overall metabolite differences between groups of samples and the magnitude of variability between samples within groups. Later, orthogonal partial least squares-discriminant analysis (OPLS-DA) and variable importance in projection (VIP) were used to screen differential metabolites in different supernatant samples, in addition to *p*-value and fold-change. The criteria were Log2FC ≥ 2 and Log2FC ≤ 0.5 or VIP ≥ 1.

### 2.6. Joint analysis of transcriptome and metabolome

Joint analysis of the data was carried out in the Metware cloud platform. The data of Groups X, Y, and Z in the transcriptome and Groups A, B, and D in the metabolome were chosen for joint analysis. These were named A (Z with A), B (X with B), and D (Y with D).

### 2.7. RT-qPCR analysis

The centrifuged bacterial sediment was ground with liquid nitrogen to extract total RNA, and the total RNA that was not significantly degraded by electrophoresis (the brightness of the 23S band was more than 1.5 times that of the 16S band) was reverse transcribed into cDNA. RT-qPCR was conducted in a 20-μL volume containing 1 μL of diluted cDNAs, 0.4 μL of the forward primer, 0.4 μL of the reverse primer, and 2 × ChamQ SYBR qPCR Master Mix (Vazyme) under the following conditions: 95 °C for 30 s, followed by 40 cycles of 95 °C for 10 s, and 60°C for 30 s using the instrument's default melting curve acquisition program (primer and sample concentrations are shown in Supplementary Tables 1, 2). The 2^−ΔΔCt^ method was used to calculate the relative expression levels (Livak and Schmittgen, 2001).

### 2.8. Statistical analysis

Three replicate trials were carried out for each sample, and data were generally expressed as the mean ± standard error (SE) in addition to other interpretations in the experiment. Data with p < 0.05 were identified as significantly different.

## 3. Results

### 3.1. Formation of suspended aggregates

**Figure 1:**
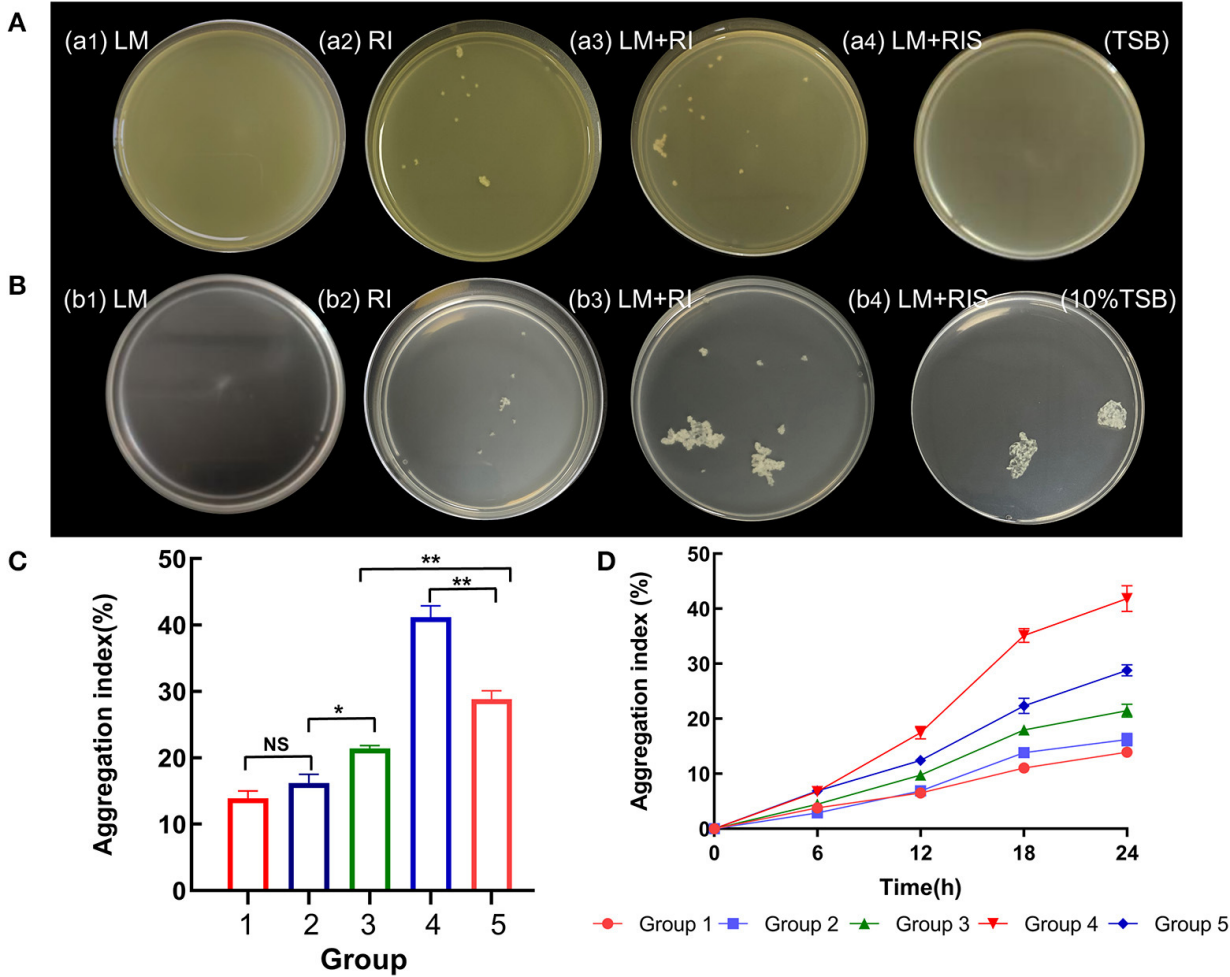
Formation of different suspended aggregates and changes of aggregation index. **(A)** Aggregates formed by bacteria cultured in tryptic soy broth (TSB); **(B)** Aggregates formed by bacteria cultured in 10% TSB; **(C)** The aggregation index of different groups; **(D)** Change in aggregation index every 6 h. 1–5 in **(C, D)** are *R. insidiosa* in TSB, *R. insidiosa* in 10% TSB, *R. insidiosa* with *L. monocytogenes* in TSB, *R. insidiosa* with *L. monocytogenes* in 10% TSB, and *L. monocytogenes* in 10% supernatant of *R. insidiosa*, respectively. *P < 0.05, **P < 0.01; NS, means not significant.

As shown in Figures 1A, B, by itself, *L. monocytogenes* could not form suspended aggregates in different nutrient concentrations of TSB alone, while *R. insidiosa* could form obvious white flocculent particle aggregates. *L. monocytogenes* co-cultured with *R. insidiosa* were able to form suspended aggregates in nutrient-poor environments, which were bigger and tighter than in normal environments. Moreover, the supernatants of *R. insidiosa* were able to induce *L. monocytogenes* to form aggregates in 10% TSB, whereas aggregates were unable to form under TSB conditions. The aggregates could be clearly seen to be connected by distinct filaments. *L. monocytogenes* co-cultured with *R. insidiosa* in 10% TSB could obtain the largest aggregation index (Figure 1C) with 41.2%, and the aggregation indices of other bacteria were 28.9, 21.4, 16.2, and 13.9%. The aggregation formation curves were plotted (Figure 1D), and it can be easily seen that the slopes of the curves were all low in 0–6 h, while in 6–18 h, especially in 12–18 h, the slopes of the curves increased significantly, indicating that strong aggregation of aggregates occurred, and in 18–24 h, the slopes of the curves decreased.

### 3.2. Microscopic examination of the suspended aggregates

**Figure 2:**
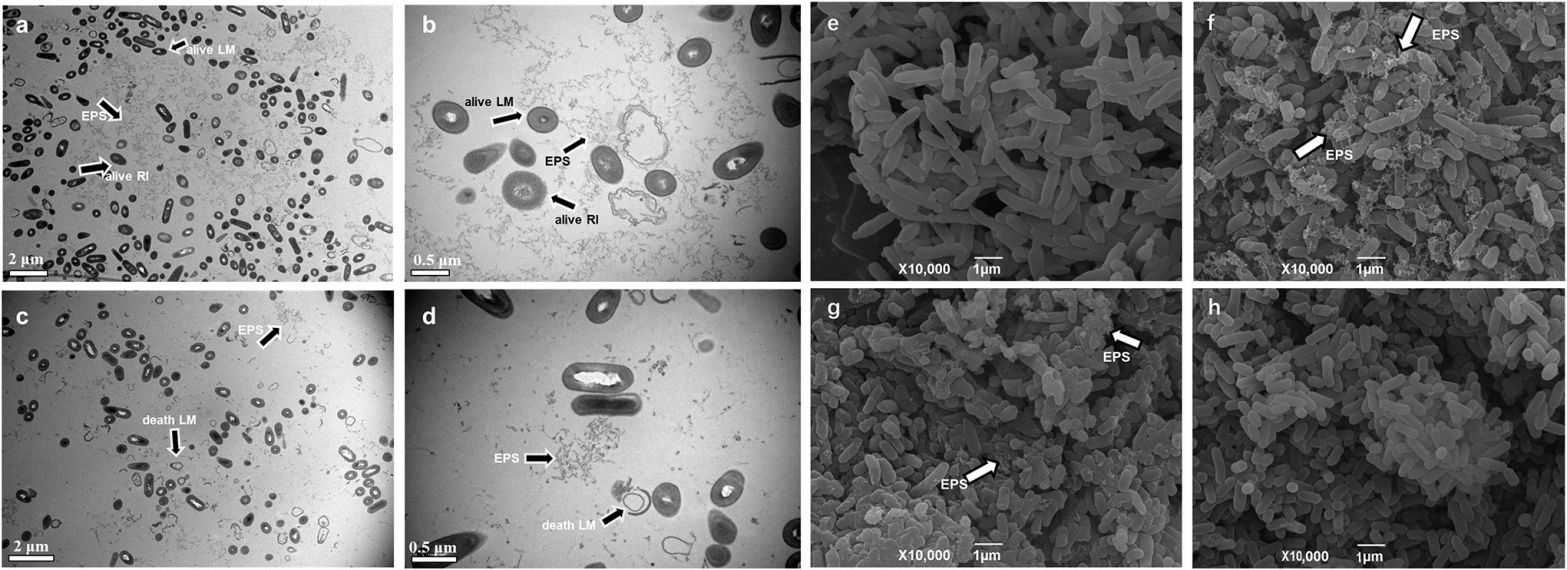
TEM and SEM images of suspended aggregates. **(a, b)** TEM of the aggregates formed by *R. insidiosa* and *L. monocytogenes* in 10% TSB with different scales; **(c, d)** TEM of the aggregates formed by *L. monocytogenes* with RIS in 10% TSB with different scales; **(e)** SEM of the planktonic bacteria *R. insidiosa* in 10% TSB; **(f)** SEM of the aggregates formed by *R. insidiosa* in 10% TSB; **(g)** SEM of the aggregates formed by *R. insidiosa* and *L. monocytogenes* in 10% TSB; **(h)** *L. monocytogenes* with RIS in 10% TSB.

The aggregates obtained from the culture of *L. monocytogenes* with *R. insidiosa* and RIS under nutrient-poor conditions (10% TSB) were observed using TEM. When *R. insidiosa* was co-cultured with *L. monocytogenes*, more bacteria were present in clusters in the microscope field of view (Figures 2a, b). When *L. monocytogenes* were co-cultured with *R. insidiosa*, both bacteria were observed to be present in the aggregates, a process in which the bacteria exerted their self-aggregation as well as co-aggregation and interactions between the strains, resulting in the formation of more pronounced and denser aggregates. The cells produced distinct and dense EPS components that were distributed around the bacteria surrounding and connecting them, thereby providing more space for their survival. In contrast, the aggregates produced by *L. monocytogenes* induced by RIS had a disorganized distribution of bacteria with cytoplasmic components spilled from dead cells and fewer EPS components with an irregular distribution (Figures 2c, d).

As shown in the SEM figures, in the nutrient-poor environment (10% TSB), the planktonic bacteria of *R. insidiosa* in the liquid environment have a smooth surface and are evenly and loosely distributed, with an overall rod shape (Figure 2e). Meanwhile, the suspended aggregates formed by *R. insidiosa* are connected by their own secreted EPS components, and as seen in the image, the surface of the bacteria has many EPS (arrow) connected clusters that wrap around the bacteria to connect them (Figure 2f). When *R. insidiosa* was co-cultured with *L. monocytogenes*, we could observe more clearly that the bacteria were more tightly connected and distributed in clusters, which had more EPS components and fewer planktonic bacteria (Figure 2g). In addition, the structure of *L. monocytogenes* aggregates induced by RIS was sparser, connected by fine filaments, and not tightly structured (Figure 1). As reflected by electron micrographs (Figure 2h), some *L. monocytogenes* aggregates were clustered but remained partially free, and their EPS had fewer components than those in the other groups. Comparing the EPS content across all groups (Figures 2f–h), it is evident that the abundant EPS content in the aggregates co-cultured by dual strains may have originated more from *R. insidiosa*.

### 3.3. Transcriptomic analysis

#### 3.3.1. Screening for differentially expressed genes

**Figure 3:**
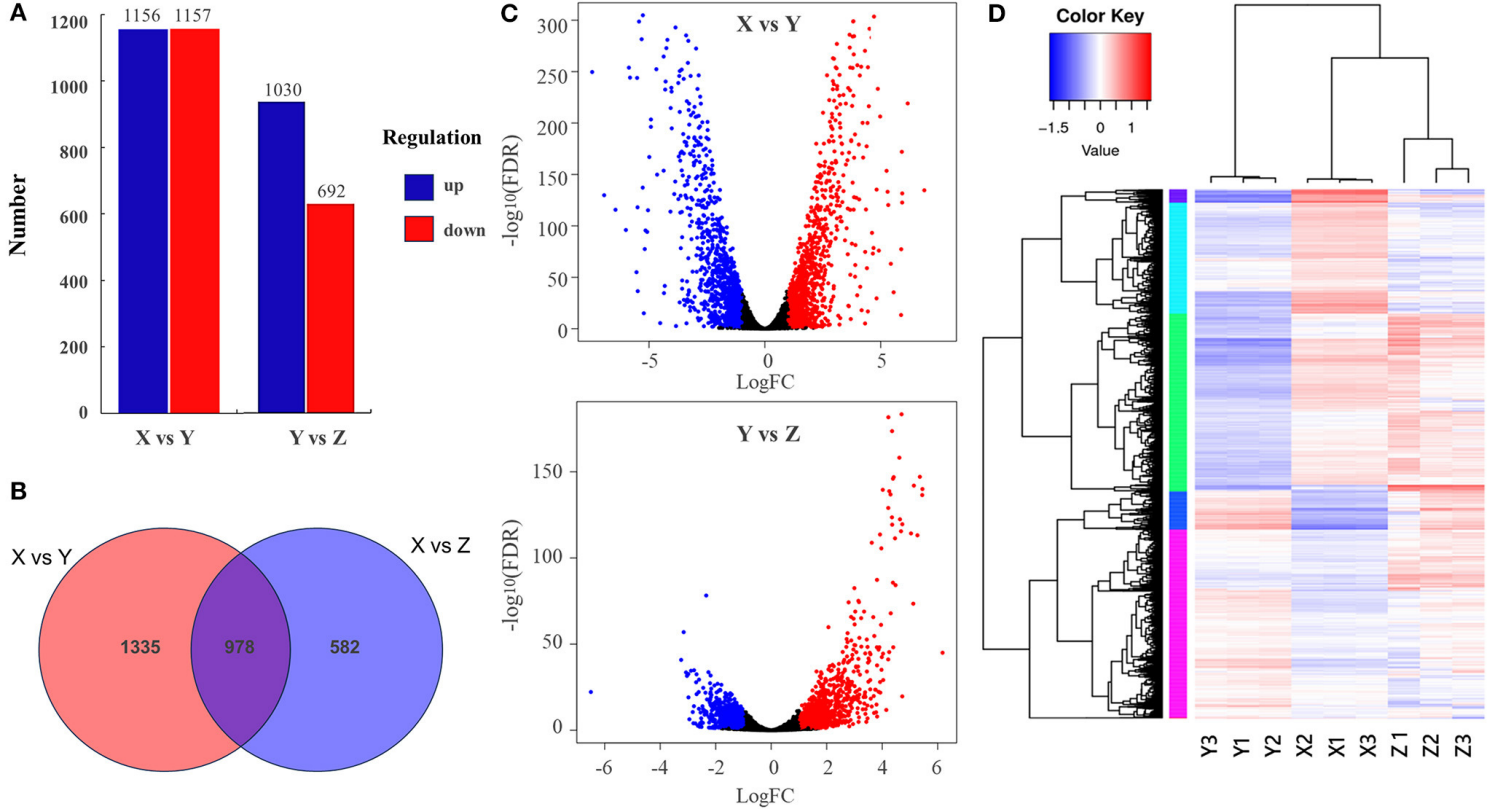
Changes in DEGs expression. **(A)** Upregulation and downregulation of DEGs; **(B)** Venn diagram showing the co-regulation of DEGs in all comparison groups; **(C)** Volcano map of differentially expressed genes (significantly different genes with red notes indicate upregulation and blue dots indicate downregulation; the abscissa represents the fold change in gene expression in different samples, and the ordinate represents the statistical significance of the differences in gene expression); **(D)** Heatmaps of DEGs compared between different groups. Sample information: X was extracted from aggregates of *R. insidiosa* in TSB, Y was extracted from aggregates of *R. insidiosa* in 10% TSB, and Z was extracted from aggregates of *R. insidiosa* co-cultured with *L. monocytogenes* in 10% TSB.

After removing the low-quality reads (contamination and joint sequences), a total of 342,295,356 clean reads were obtained. The percentages of Q30 (percentage of a total number of bases with Phred values≧30) and GC were 93.85–95.7% and 42.94–61.69%, indicating that the quality of transcriptome data was high. According to the specifically mapped reads, FPKM values were calculated for the evaluation of relative gene expression levels, and the DESeq2 software was utilized to explore the differences in gene expression. The gene significant differential expression was screened and counted according to the rules of Log_2_FC≥2 and p ≤ 0,05. The results are shown in Figure 3. The highest number of DEGs was detected when comparing TSB with 10% TSB (X vs. Y), and the same results are shown in the heat map (Figures 3B, D). Among them, TSB vs. 10% TSB (X vs. Y) and RI vs. RI with LM (Y vs. Z) were compared, and 2,313 (1,156 upregulated and 1,157 downregulated) and 1,722 (1,030 upregulated and 692 downregulated) significant genes were identified, respectively (Figure 3A), indicating that the formation of suspended aggregates was accompanied by changes in transcripts. Eight significantly enriched pathways were screened for p ≤ 0.05, and the gene with the largest Log_2_FC was selected in each pathway. The screened pathways included the “Metabolic pathways,” “Phenylalanine metabolism,” etc., in the transcriptome data, and these genes were used as targets for RT-qPCR assays.

#### 3.3.2. GO enrichment analysis of differentially expressed genes

**Figure 4:**
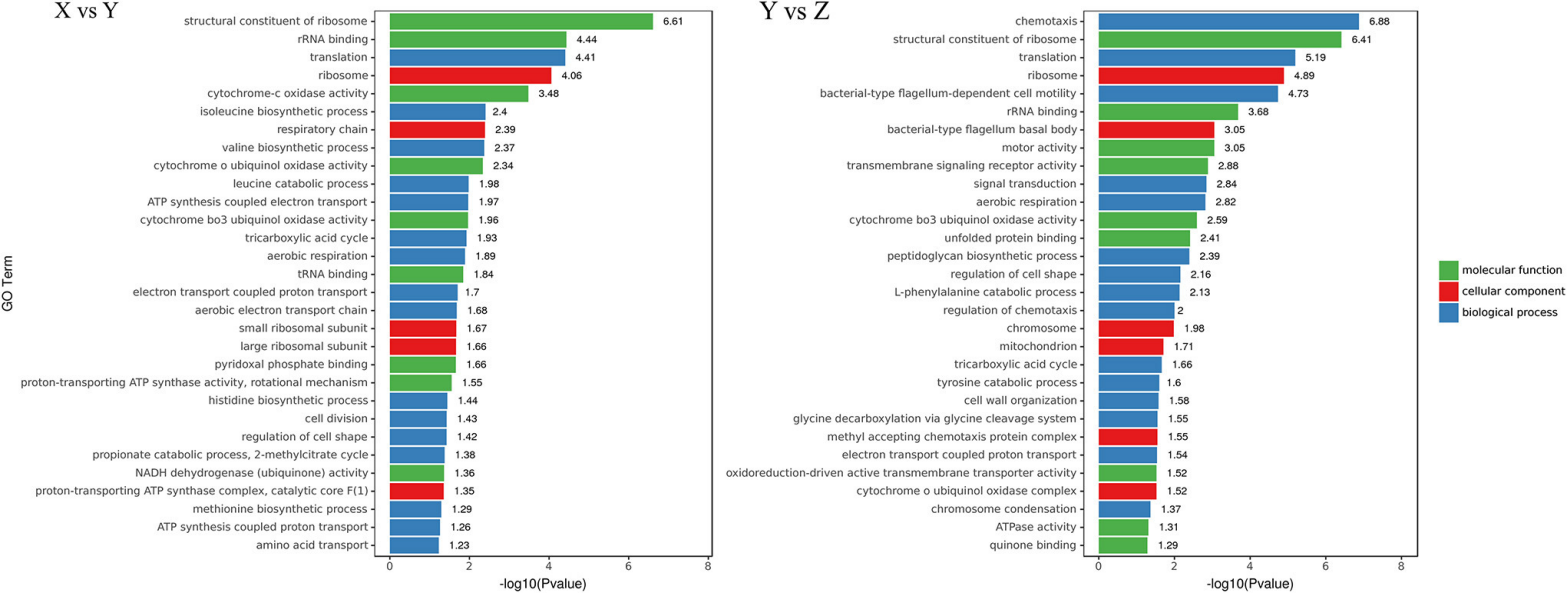
Gene Ontology (GO) distribution map of differentially expressed genes (DEGs) in three main categories. The ordinate is the GO term, and the abscissa is the log_10_(*p*-value) of DEGs in the term. X vs. Y is TSB vs. 10% TSB; Y vs. Z is RI vs. RI with LM.

To further evaluate the biological functions of DEGs in suspended aggregate formation, the GO annotations were categorized after obtaining the significant DEGs to visualize the GO function distribution characteristics of the DEGs (Figure 4). The results showed that the most obvious enriched terms in three ontologies—“molecular function,” “cellular component,” and “biological processes”—were “structural composition of ribosome” (GO:0003735), “ribosome” (GO:0005840), and “translation” (GO: 0006412) in the TSB vs. 10% TSB (X vs. Y) group, and “structural composition of ribosome” (GO:0003735), “ribosomes” (GO:0005840), and “chemotaxis” (GO:0006935) in the RI vs. RI with LM (Y vs. Z) group. This suggests that “ribosomes” may mainly be involved in the formation of suspended aggregates in barren environments and may generate amino acids and proteins through translation and influence the formation of aggregates through amino acids and other metabolic pathways.

#### 3.3.3. KEGG enrichment analysis of differentially expressed genes

**Figure 5:**
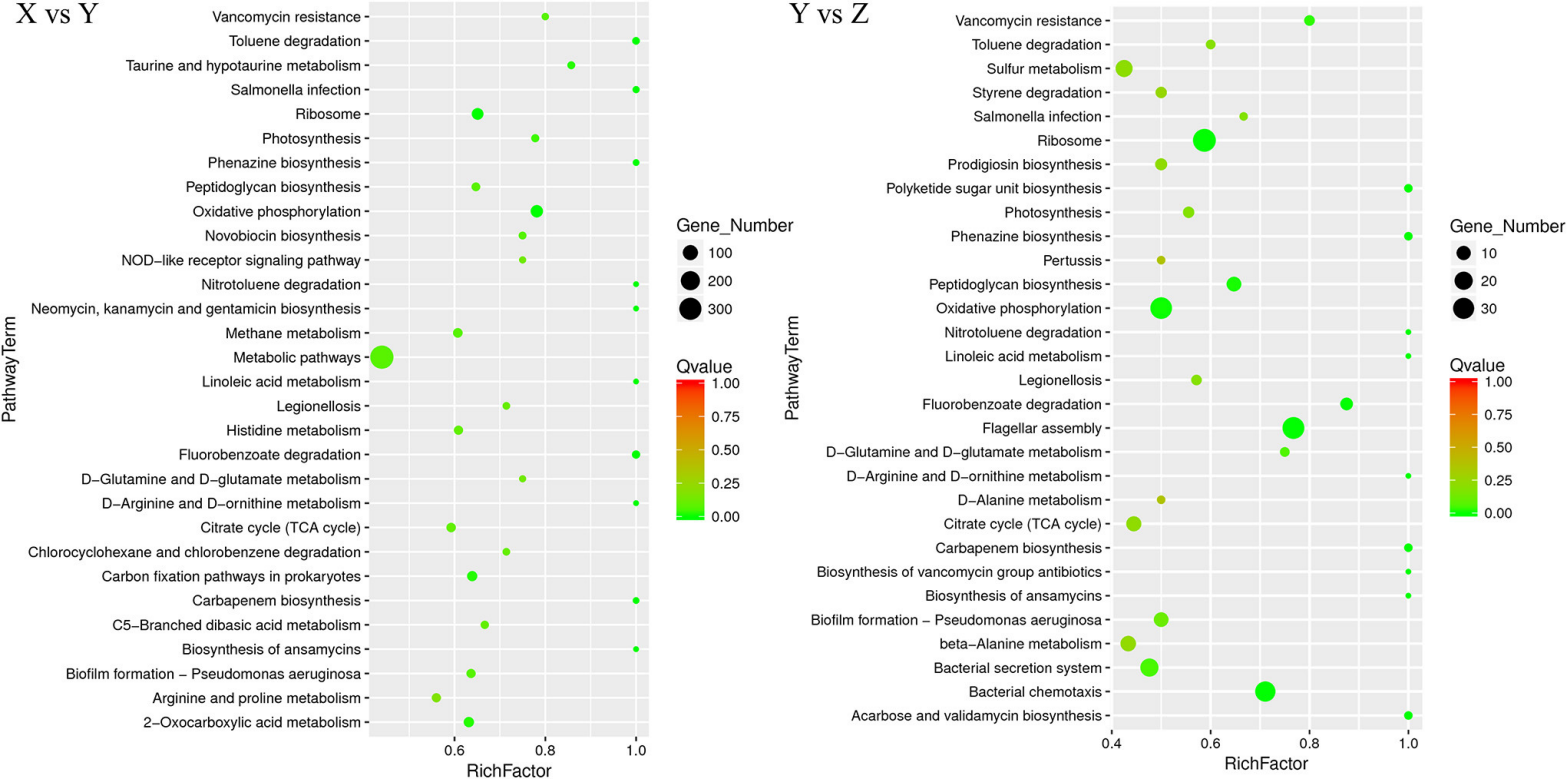
Scatter plot of Kyoto Encyclopedia of Genes and Genomes (KEGG) pathway enrichment for DEGs. The number of DEGs in each pathway is closely related to the size of dots, and the color of dots reflects different q values. The rich factor was positively correlated with the enrichment degree. The smaller the q-value, the more significant the enrichment. X vs. Y is TSB vs. 10% TSB; Y vs. Z is RI vs. RI with LM.

The KEGG enrichment pathways of DEGs are shown in Figure 5. Between the TSB vs.10% TSB (X vs. Y) groups, there were 24 M (metabolic) pathways, 3 HD (human disease) pathways, 1 GIP (genetic information processing) pathway, 1 OS (biological systems) pathway, and 1 CP (cellular processes) pathway. The top 5 enriched pathways related to metabolism were “D-Arginine and D-ornithine metabolism,” “toluene degradation,” “oxidative phosphorylation,” “carbon fixation pathways in prokaryotes,” and “2-oxocarboxylic acid metabolism.” The pathway related to GIP was “ribosome,” and the pathway related to CP was “biofilm formation.” In the RI vs. RI with LM (Y vs. Z) group, there were 21 M pathways, 4 HD pathways, 1 GIP pathway, 1 EIP (environmental information processing) pathway, and 3 CP pathways. The top 4 enriched pathways related to metabolism were “linoleic acid metabolism,” “oxidative phosphorylation,” “peptidoglycan biosynthesis,” and “D-glutamine and D-glutamate metabolism.” The pathways related to GIP were “ribosomes,” and the main pathways related to CP were “flagellar assembly” and “bacterial chemotaxis.” Those related to EIP were “bacterial secretion system.”

#### 3.3.4. RT-qPCR verification of gene expression

To confirm the accuracy of the genes obtained by RNA seq, we randomly selected 9 DEGs belonging to different metabolic pathways and other pathways (Table 1). In addition to gene *serA*, the similar expression trend of selected DEG was consistent with that of Illumina sequencing, indicating the reliability of RNA-Seq data (Supplementary Figure 1). The 16S gene of *R. insidiosa* served as the reference gene (detailed data information can be seen in Supplementary Table 3).

**Table 1:** Regulation of differential genes in transcriptome data.

**Genes**	**KO_name**	**Pathways**	**Log**_**2**_ **foldchange**		
			**X vs.Y**	**X vs.Z**	**Y vs.Z**
Gene4182	*echA*	Phenylalanine metabolism	—	2.14	2.18
Gene4606	*qseB*	Two-component system	2.50	2.66	—
Gene5232	*serA*	Glycine, serine, and threonine metabolism	1.36	1.17	−1.27
Gene3579	*metQ*	ABC transporters	1.92	1.71	—
Gene5140	*acd*	Carbon metabolism	−4.91	-	4.39
Gene4092	*metE*	Cysteine and methionine metabolism	6.32	4.66	−1.49
Gene4973	*coxA*	Metabolic pathways	4.06	3.49	3.77
Gene4057	*E4.4.1.11*	Cysteine and methionine metabolism	2.98	—	−2.09
Gene1941	*hydA*	2-oxocarboxylic acid metabolism	1.86	3.42	1.59

### 3.4. Metabolomic analysis

#### 3.4.1. Quality control of metabolomic data

**Figure 6:**
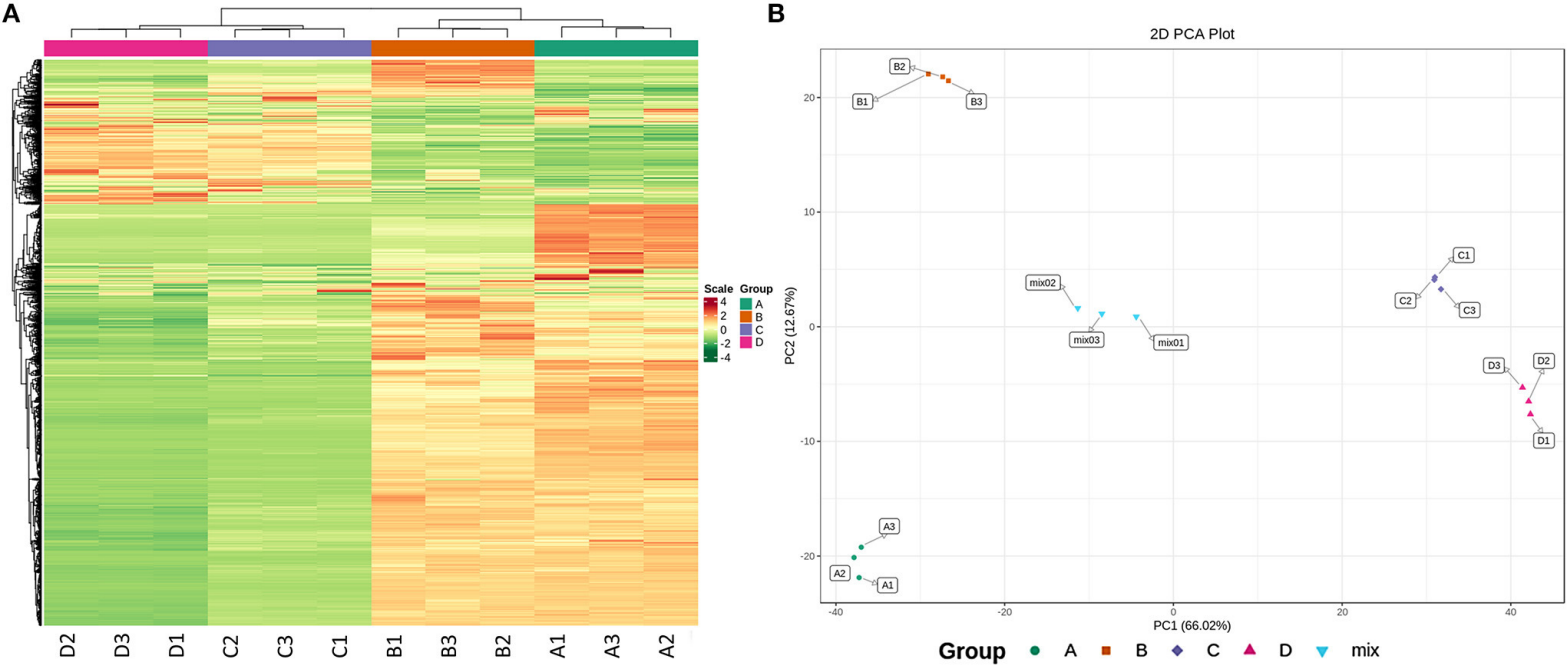
Quality control of metabolomics data. **(A)** Heatmap showing the results of the clustering analysis of DAMs (horizontal: sample information, vertical: metabolite information, scale: the value of metabolite relative content after standardized processing; the redder the color is, the higher the content); **(B)** PCA score of DAMs (horizontal and vertical axes refer to the first and second principal components, respectively, and percentages; the values contributed by the principal components to the sample differences; the same color means the same component). A is TSB without inoculation; B is RIS of TSB; C is RIS of 20% TSB; D is RIS of 10% TSB.

In this part of the study, TSB was used as a control group to explore the metabolites of *R. insidiosa* under different nutritional conditions, and their contents were normalized to construct a hierarchical clustering heat map (Figure 6A). Subsequent principal component analysis (PCA) score plots concerning the overall sample (Figure 6B) showed a significant separation of metabolic components among the groups. Orthogonal projection-discriminant analysis (OPLS-DA) was performed on the potential structures, and the scores showed that the different treatment groups also showed significant separation, indicating that the data analysis results for each sample were accurate and differed significantly between groups.

#### 3.4.2. Metabolite content analysis

**Figure 7:**
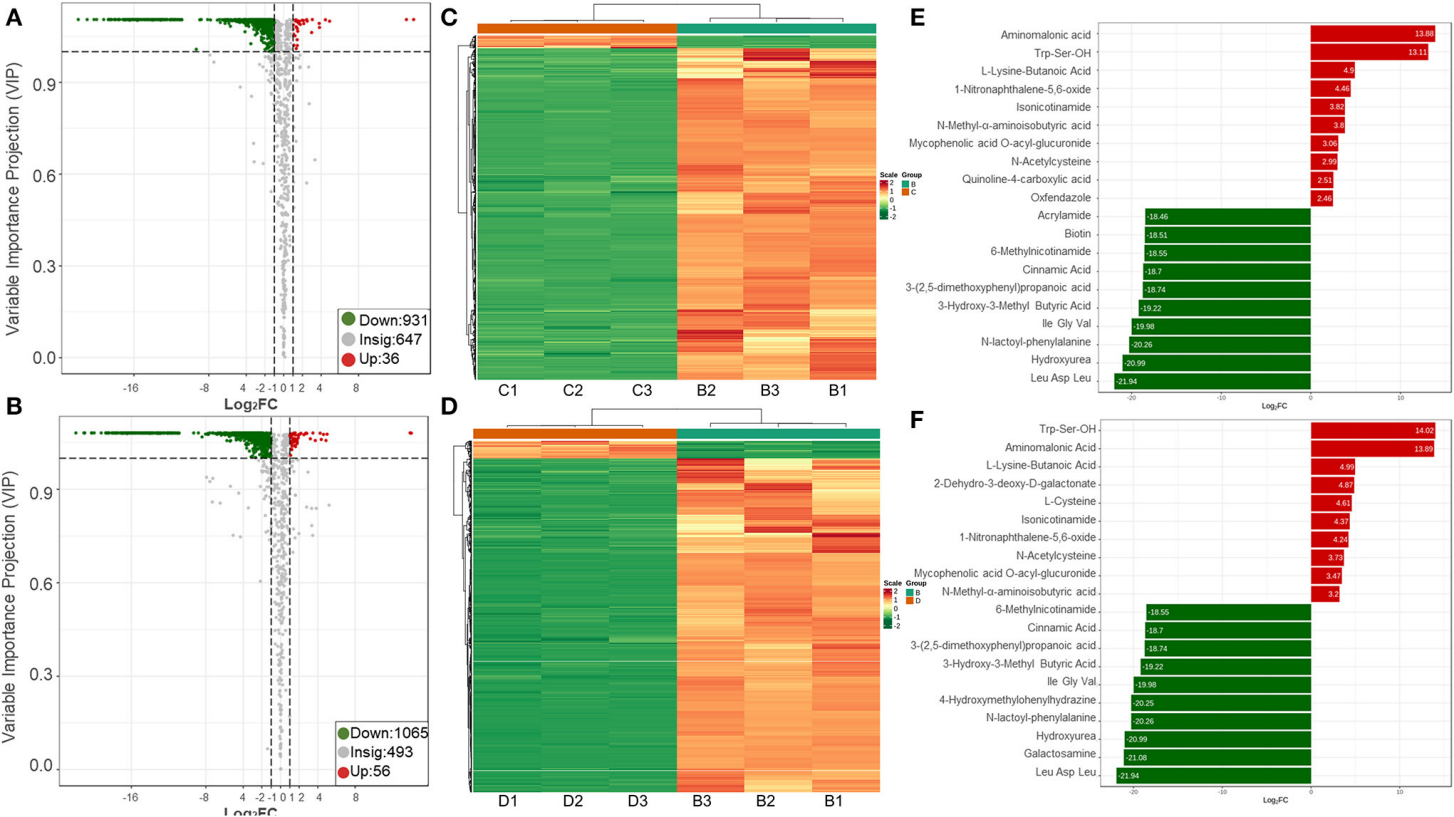
Upregulation and downregulation of DAMs in different treatment groups. **(A, B)** Volcano plots of the regulated metabolites; **(C, D)** Heatmaps of the regulated metabolites; **(E, F)** Variations of the top 10 DAMs. The first row is the B vs. C group, and the second row is the B vs. D group. B vs. C is RIS of TSB vs. RIS of 20% TSB; B vs. D group is RIS of TSB vs. RIS of 10% TSB.

Sterile supernatants from *R. insidiosa* in 20% TSB and 10% TSB induced suspended aggregates in *L. monocytogenes*, but not TSB. Cluster analysis based on multivariate statistics was performed to easily and directly detect these differences in metabolites. The different or similar significant metabolites with Log_2_FC ≥ 2 or Log_2_FC ≤ 0.5 and VIP ≥ 1 were selected for further analysis using volcano plots (Figures 7A, B). We screened a total of 56 upregulated components and 1055 downregulated components in TSB vs. 10% TSB (B vs. D), of which the details of the upregulated components are shown in Supplementary Table 4. The 56 significantly different metabolites included 18, 12, 4, and 3 amino acids and their metabolites, organic acids and their derivatives, phenols, and carbohydrates and their metabolites, respectively. Among them, the aminomalonic acid and Proximicin B were upregulated 15,000 and 8,000 times in Groups C (20% TSB) and D (10% TSB), respectively. However, the relative contents in Group B (TSB) were 0, indicating that these two components were unique and critical. A total of 31 components in TSB vs. 20% TSB (B vs. C) were similar to the differential metabolites of TSB vs. 10% TSB (B vs. D), mainly including amino acids and their metabolites, organic acids and their derivatives, and phenolic components, which accounted for the majority of 6, 6, and 4, respectively. The heat map of DAMs was plotted, and the DAMs of these two control groups reflected the above changes (Figures 7C, D). Based on the fold change in metabolite accumulation, we identified the top ten DAMs that increased or decreased in each control group (Figures 7E, F).

#### 3.4.3. Metabolic pathway analysis of differential metabolites

**Figure 8:**
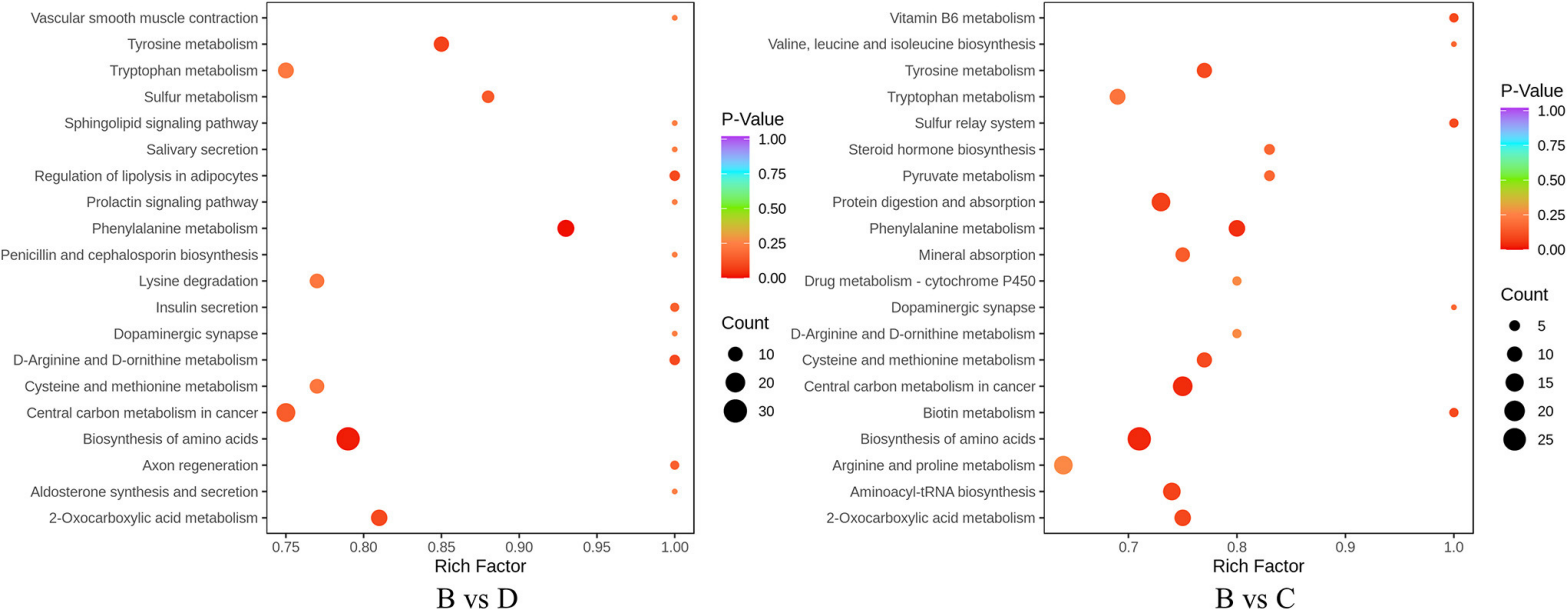
Different metabolite KEGG enrichment maps. The horizontal axis is the Rich factor corresponding to different pathways, the vertical axis is the name of the pathway, the shade of the color is proportional to the degree of enrichment, and the area of the dots reacts to the number of differential metabolites. B vs. C is RIS of TSB vs. RIS of 20% TSB; B vs. D group is RIS of TSB vs. RIS of 10% TSB.

The annotation and pathway enrichment of differential metabolites were used by KEGG. There were 196 and 220 components in the TSB vs. 20% TSB (B vs. C) and TSB vs. 10% TSB (B vs. D) groups of differential metabolites that were annotated to different metabolic pathways (Figure 8). The four pathways with the highest enrichment significance in both groups were “biosynthesis of amino acids,” “2-oxocarboxylic acid metabolism,” “phenylalanine metabolism,” and “tyrosine metabolism.” In addition, “amino acid and aminoacyl tRNA biosynthesis,” “phenylalanine metabolism,” and “protein digestion and absorption” were the major metabolic pathways in TSB vs. 20% TSB (B vs. C), with the number of differential metabolites being 38, 19, 15, and 22. “Phenylalanine metabolism,” “tyrosine metabolism,” and “biosynthesis of amino acid” are the major metabolic pathways in TSB vs. 10% TSB (B vs. D), with the number of differential metabolites being 15, 13, and 38. Two groups of differential metabolites were mainly enriched in pathways related to amino acid synthesis and metabolism.

### 3.5. Joint analysis of transcriptome and metabolome

#### 3.5.1. Correlation between genes and metabolites

**Figure 9:**
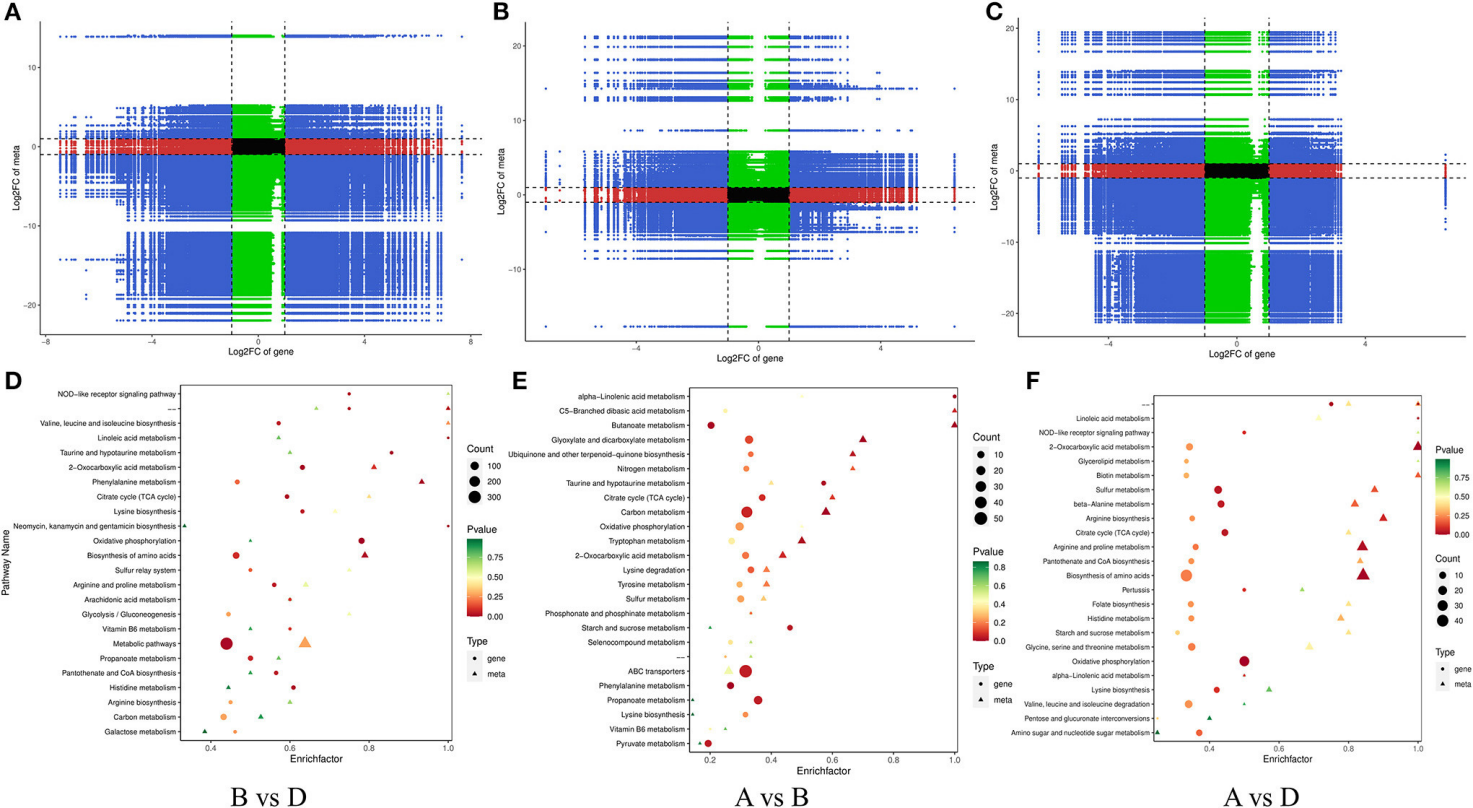
Correlation analysis of transcriptomic and metabolomic. **(A–C)** The nine-quadrant diagram: horizontal coordinates represent the log_2_FC of genes, and vertical coordinates represent the log_2_FC of metabolites; **(D–F)** The bar charts of KEGG enrichment analysis. The horizontal coordinate represents the rich factor of differential metabolites and DEGs enriched to the pathway, and the vertical coordinate represents the name of the KEGG pathway.

The transcriptome and metabolome were jointly analyzed to further understand the possible metabolic pathways of *L. monocytogenes* aggregates formation induced by *R. insidiosa*. The correlation analysis of the nine-quadrant plot (Figure 9A) shows that many genes showed a strong positive correlation (R > 0.8) with metabolites and that changes in the accumulation of these metabolites may be directly or indirectly regulated by the corresponding genes.

In the TSB vs.10% TSB (B vs. D) group, the pathways with key differential metabolites (*p* < 0.01) were phenylalanine metabolism (ko00360), biosynthesis of amino acids (ko01230), Tyrosine metabolism (ko00350), and 2-oxocarboxylic acid metabolism (ko01210). Pathways with key difference genes (*p* < 0.01) were linoleic acid metabolism (ko00591), oxidative phosphorylation (ko00190), 2-oxocarboxylic acid metabolism (ko01210), and taurine and hypotaurine metabolism (ko00430). In the RI vs. RI with LM (A vs. D) group, the pathways with key differential metabolites (*p* < 0.01) were 2-oxocarboxylic acid metabolism (ko01210), phenylalanine metabolism (ko00360), cysteine and methionine metabolism (ko00270), and biosynthesis of amino acids (ko01230). Pathways with key difference genes (*p* < 0.01) were linoleic acid metabolism (ko00591), oxidative phosphorylation (ko00190), sulfur metabolism (ko00920), citrate cycle (ko00020), and beta-alanine metabolism (ko00410).

#### 3.5.2. Effect of nutritional conditions on metabolites

The alteration and accumulation of components such as amino acids play a key role in the induction response of *R. insidiosa* to form aggregations when the transcriptome analysis is combined with metabolome analysis data (Figure 9). Therefore, in this study, we focused on amino acids and other components detected at low nutrient concentrations. When comparing groups with different nutritional conditions, amino acid biosynthesis and metabolism of different amino acids were the most significantly enriched, which also interrelate with multiple genes (Supplementary Table 6). Combining the results of single transcriptomic and metabolomic groups, this conclusion was better verified by the direct or indirect involvement of ribosomes in the synthesis and metabolism of amino acids when *R. insidiosa* was cultured under lower nutrient conditions. This significantly affected the expression of *R. insidiosa* and amino acid-related genes and the accumulation of metabolites, changing the metabolic composition of the bacterial supernatant and thus inducing the formation of suspended aggregates in *L. monocytogenes*.

## 4. Discussion

The coexistence and growth of multiple microorganisms in different environments, interacting with each other to form suspended aggregates, increases the resistance of microorganisms to environmental stress. In recent years, environmental bacteria have been widely prevalent in food processing environments, boosting the survival of various types of pathogenic bacteria (Ricci et al., 2018). Among them, the presence of *R. insidiosa* in food processing plants in a low-nutrient environment after cleaning and processing can lead to long-lasting contamination and survival of *L. monocytogenes*, posing a great threat to food safety (Li et al., 2021).

The results from the incubation of different bacteria under different conditions were similar to those from a previous study (Chen et al., 2021). However, the difference was that incubating the bacteria under optimized conditions (reduced speed) resulted in the formation of more pronounced and dense particles. In addition, monitoring the aggregates every 6 h allowed a closer inspection of the formation of aggregates from a temporal perspective. During the first 6–12 h, bacteria tend to form the initial type of aggregates. Then, between 12 and 18 h, they adhere and aggregate heavily on this initial foundation. Therefore, it can be concluded that the critical period for the formation of aggregates is from 6 to 18 h. The environmental bacterium *R. insidiosa* has good survivability and is able to form suspended aggregates under conditions of poor nutrition and low speed. This may be because nutritional deficiencies push *R. insidiosa* to cluster together during growth and reproduction, thereby reducing the need for nutrients to better withstand nutritional stress. While *L. monocytogenes* cannot form aggregates on their own in liquid environments, the presence of *R. insidiosa* and RIS (acquired from 20% TSB and 10% TSB) could promote *L. monocytogenes* to form the suspended aggregates; the promotion effect was quite obvious in our study. This suggests that the process of aggregate formation is related to not only the interactions between the dual strains but also the essential metabolites of *R. insidiosa* when culturing in a nutrient-poor environment (Laganenka et al., 2016).

The ultrastructure of the aggregates was successfully observed using transmission and scanning electron microscopy. It was found that EPS were present around the bacteria that formed the aggregates, among which the aggregates formed by *R. insidiosa* had the highest concentration of EPS. This indicates that the process of aggregate formation is similar to that of biofilms, for which the analysis can be based on the related studies of biofilms.

The transcriptome data of TSB vs.10% TSB (X vs. Y) groups were used to associate key DEGs with aggregate formation. From the sequenced RNA libraries, we obtained 1,031 upregulated DEGs and 692 downregulated DEGs. GO and KEGG enrichment analyses were performed based on the obtained DEGs, and 102 biochemical metabolic pathways were identified. The genes mainly associated with metabolism were oxidative phosphorylation, 2-oxocarboxylic acid metabolism, and linoleic acid metabolism, with a *p*-value of ≤0.05 as the basis. The genes associated with GIP were ribosome, and with CP were flagellar assembly, bacteria chemotaxis, and biofilm formation. When selected by gene number, they were mainly associated with metabolic pathways, biosynthesis of secondary metabolites, two-component systems, and amino acid biosynthesis. In the nutrient-poor environments, *acdx, puuE*, and *acs* genes of *R. insidiosa* were significantly upregulated in biosynthetic processes such as carbon metabolism, metabolic pathways, and biosynthesis of secondary metabolites, with Log_2_FC reaching 4.39, 3.96, and 3.95, respectively, while the Log_2_FC of *cydA, cyoB*, and *rpsJ* in oxidative phosphorylation and ribosomal pathways reached 3.74, 3.87 and 4.25, respectively. Among them, *cyo* and *cox* family genes were strongly involved in metabolic pathway processes and were connected to the two-component system through *regA*. The chemotactic proteins *cheA* and *cheY* shuttled back and forth between the receptor complex and flagellar motor complex and were involved in ABC transport and quorum sensing processes through the *flh*D gene in conjunction with the ribosome binding site (*rbs*).

Co-aggregating species often promote competition or synergy between each other through the production of cellular signals, metabolite exchange, or bacteriocins and rely on a series of interactions (Anand et al., 2022). During the formation of aggregates, microorganisms display different phenotypes and perform different functions (Fagerlund et al., 2017). When aggregates are formed, they are generally encapsulated with a large number of EPS components, which contain a variety of functional groups that protect the cells inside through complexation and valence (Gu et al., 2018). *R. insidiosa*, under nutrient-poor stress, produced specific products through a variety of pathways, including oxidative phosphorylation. These induced *L. monocytogenes* to adjust sigma signaling molecules and sensors of two-component systems, etc., and form a tightly knit cluster. This had the potential to survive with common outward acquisition capabilities by generating population sensing and flagellar assembly.

The supernatants of *R. insidiosa* cultured in 20% TSB (B vs. C) and 10% TSB (B vs. D) induced *L. monocytogenes* to form aggregates, so we focused on similar components between both groups. Among the 31 similar metabolites common to both groups (Supplementary Table 4), aminomalonic acid and Proximicin B were the components with the highest upregulation fold, specific to both groups. Aminomalonic acid is a naturally occurring non-protein-derived amino acid that may originate from protein synthesis errors and oxidative damage to protein amino acid residues (Voinova et al., 2020), or it may be an oxidation product resulting from disturbances in serine and threonine metabolism (Copley et al., 1992), and its malonic acid fraction may incubate protein calcium-binding properties (Park and Crawford, 2015). Co-aggregation between different prototype strains is usually mediated by aggregate-sugar interactions, i.e., strain cell surface-associated lectin-like proteins that recognize and bind to complementary polysaccharide receptors on the cell surface of the partner species (Stevens et al., 2015). The aggregates of *Lactobacillus* and *Helicobacter pylori* were fractionally isolated, and it was found that a 40 ku-50 kU surface protein was the potential material basis for the aggregates, while the strains with bigger aggregates of different *Lactobacillus* strains had more significant metabolic and amino acid transporter genes (Li et al., 2022). Therefore, the formation of aggregates may be indirectly related to specific proteins that later undergo metabolism to specific small molecules to induce the process.

The joint analysis of transcriptome and metabolome enabled the screening of relevant genes and pathways in terms of metabolic pathways and key metabolites. Following the co-enrichment analysis, 66 metabolic pathways were screened, most of which were related to amino acid metabolism, phenylalanine metabolism, oxidative phosphorylation, and the citric acid cycle. Among them, gene clusters *arg, trp, glt, leu, dap, his*, etc., were involved in amino acid metabolism and transport.

During the formation of biofilms, carbohydrate metabolism, lipid metabolism, amino acid metabolism, and nucleotide metabolism are involved and coordinated as a whole. The process mainly depends on the two-component system, QS, c-di-GMP, and cAMP, to regulate the motility of bacterial pilus and the synthesis of EPS to promote bacterial adsorption on the carrier, and by promoting the synthesis of threonine or inhibiting the synthesis of galactose (Liu et al., 2021a). Moreover, bacteria could enhance the cation transport and produce metabolisms like glyoxylate, dicarboxylic acids, and peptidoglycan to promote biofilm growth and repair cells by Lux C (Liu et al., 2021b). The formation of aggregates has some metabolic processes similar to biofilms. Aggregates share similar formation pathways with biofilms, such as amino acid metabolism (serine, glycine, lysine, etc.), enhanced energy supply pathways such as oxidative phosphorylation and the TCA cycle, in addition to a trend of upregulation of genes related to glycolysis, carbon metabolism, and metabolic pathways (Wang et al., 2021). The formation of aggregates by dual strains also interacted with each other through quorum sensing, flagellar assembly, chemotaxis, and two-component systems. However, the DEGs for aggregate still differed from those of biofilm, including cyclic adenosine monophosphate (cAMP) and bis-(3′-5′)-cyclic diguanosine monophosphate (c-di-GMP). Biofilm formation would decrease under nutrient stress, but conversely, the index of aggregates would increase under these conditions. In addition, it was found that mutations that inhibit biofilms do not prevent bacteria from forming aggregates (Staudinger et al., 2014). This suggests that despite some similarities, there are differences between the formation of aggregates and biofilm, and the two formation processes are not identical.

**Figure 10:**
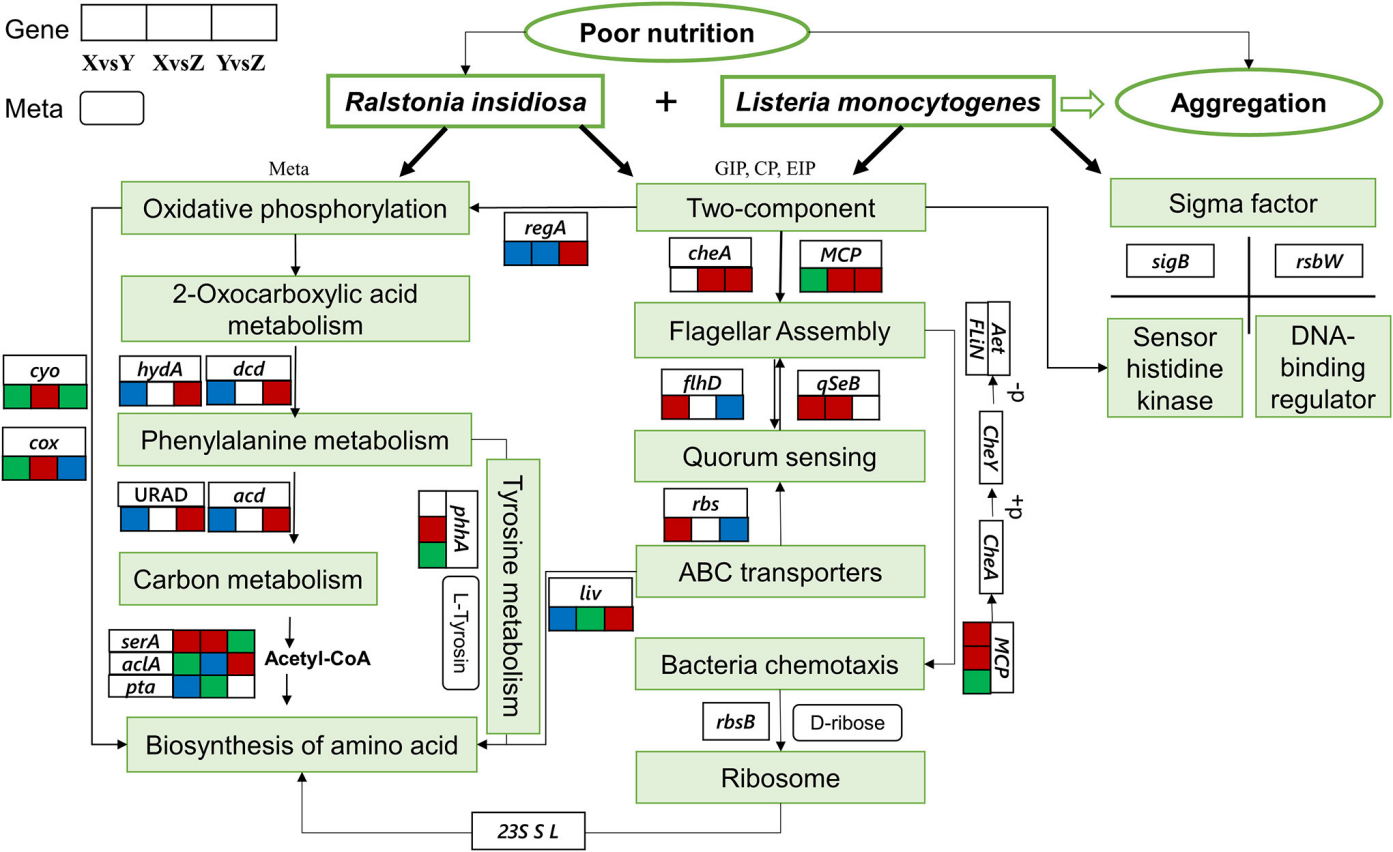
Gene regulation of the formation of the suspended aggregates. The three connected squares represent the relevant gene changes in different groups. Red indicates upregulation, blue indicates downregulation, and green indicates both up and downregulation. Boxes with sharp corners indicate genes, and boxes with obtuse corners indicate metabolites.

From analyzing all the data, we hypothesized some important pathways for the formation of the aggregation (Figure 10). Under nutrient-deprived stress, *R. insidiosa* could produce specific metabolites, such as aminomalonic acid, Proximicin B and other amino acids, organic acids, and phenols through “oxidative phosphorylation,” “amino acid biosynthesis,” “Phenylalanine metabolism” and other pathways to induce *L. monocytogenes* to adjust the transcriptional expression of the sigma factor and the two-component system of sensor histidine kinase and DNA-binding response regulator to receives signals and regulatory information. In addition, *R. insidiosa* could transport substances through ABC transport proteins and maintain intracellular homeostasis, using quorum sensing to enhance flagellar assembly to form the large and dense aggregation structure under the action of quorum sensing and flagellar assembly. The formed aggregates survive under stressful conditions by using external nutrients for energy through various metabolic pathways such as “carbon metabolism.”

## 5. Conclusion

When bacteria are cultured in a nutrient-poor and low-speed shaking environment, their growth and physiology show more positive and comprehensive stress effects than in a normal environment. The ability of sterile supernatants obtained from *R. insidiosa* cultures in a barren environment to induce *L. monocytogenes* to form aggregates shifted our attention from dual-strain interactions to metabolite formation. Our joint transcriptomic and metabolomic analyses offered some putative clues on the mechanism of the aggregate formation processes. In nutrient-poor environments, *R. insidiosa* increases the change of components such as aminomalonic acid through oxidative phosphorylation, phenylalanine, and other pathways, especially amino acid biosynthesis and carbon metabolism. These metabolisms could generate EPS to attract nearby bacteria and allow *L. monocytogenes*, which otherwise cannot form aggregates, to form aggregates. This results in a more persistent survival and contamination of *L. monocytogenes* and poses certain potential risks to the food industry. The findings from this study provide an understanding of how *R. insidiosa* induces *L. monocytogenes* to form aggregates from the perspective of dual strain interactions and metabolites. Based on this study, further investigations can be undertaken to screen for access to metabolites, such as gene editing of differential genes, for a more in-depth and specific analysis of the process and mechanism of the formation of the suspended aggregates.

## Data availability statement

The datasets presented in this study can be found in online repositories. The names of the repository/repositories and accession number(s) can be found below: https://www.ncbi.nlm.nih.gov/, PRJNA996090.

## Author contributions

XZu: Writing—original draft, Data curation. MC: Writing—review and editing. XZh: Software, Writing—review and editing. AG: Conceptualization, Supervision, Writing—review and editing. SC: Formal analysis, Writing—review and editing. RZ: Funding acquisition, Methodology, Writing—review and editing.
