# Supplementary material for: Transcriptomic and metabolomic analyses to study the key role by which Ralstonia insidiosa induces Listeria monocytogenes to form suspended aggregates
Source: Front Microbiol. 2023 Oct 12;14:1260909. doi: 10.3389/fmicb.2023.1260909 (PMC10601645; doi:10.3389/fmicb.2023.1260909)
Supplement: Supplementary file 1 [file Data_Sheet_1.zip › Data Sheet 1.DOCX]

Supplementary Material

# Supplementary Tables

**Table S1**. Primer design for RT-qPCR

| **Gene** | **Upper Primer** | **Lower Primer** | **Length of Product** |
| --- | --- | --- | --- |
| Gene4182 | GGAATCGGCGGTCATGTAG | CTCAACGGTGTGGAAGACG | 88 bp |
| Gene4606 | GTTGGTGGTGATCCATT | TCCTCATTGAAGACGAC | 118 bp |
| Gene5232 | GACCGACACCACGCATCCGC | CAAGTCCCACTCTTCGTAG | 88 bp |
| Gene3579 | GCTTCCATCAACACCGACTA | ACGGCTGGTTCTTGTCTTTC | 130 bp |
| Gene1819 | TGGAACGTACTGTCGGCAT | TCAGGTCTTCACACAGGTCG | 225 bp |
| Gene4092 | CGGAGTTCAACGACATCATC | AATGTTCGGCGAGTGGATGT | 161 bp |
| Gene4973 | CGTACTTTGGTTTCTTCT | CACCCAGTTGTAGACCTTGA | 77 bp |
| Gene4057 | AGTCCGACATTGAGCCGCA | GTGTTCTCGGTGGTGTTTCA | 185 bp |
| Gene1941 | TCTTCACGGAAATCGTCTTG | GCATTACCTCGACCAACGCT | 126 bp |

**Table S2**. Specific experimental amplification system of RT-qPCR

| **Component** | **Group** | **Concentration** | **Volume (μL)** |
| --- | --- | --- | --- |
| cDNA | RI+TSB | 1173 ng/μL | 0.085 |
| cDNA | RI+1/10TSB | 1321 ng/μL | 0.075 |
| cDNA | RI+LM+1/10TSB | 1017 ng/μL | 0.1 |
| Prime 1 |  | 0.2 μM | 0.4 |
| Prime 2 |  |  | 0.4 |
| 2×ChamQ SYBR qPCR mix |  |  | 10 |
| ddH2O |  |  | To 20 |

**Table S3**. The Log2FoldChange of Transcription sequencing and RT-qPCR

| Gene | Pathway | 1 | | 2 | | 3 | |
| --- | --- | --- | --- | --- | --- | --- | --- |
|  |  | TS | qPCR | TS | qPCR | TS | qPCR |
| Gene4182 | Phenylalanine metabolism | — | 0.395±0.18 | 2.13 | 2.47±0.46 | 2.18 | 2.74±0.16 |
| Gene4606 | Two-component system | 2.50 | 2.20±0.31 | 2.66 | 2.18±0.15 | — | 0.30±0.27 |
| Gene5232 | Glycine, serine and threonine metabolism | 1.36 | 1.09±0.15 | 1.17 | 7.74±0.75 | -1.27 | -6.19±0.23 |
| Gene3579 | ABC transporters | 1.92 | 2.64±0.44 | 1.71 | 1.25±0.26 | — | 0.54±0.26 |
| Gene1819 | Purine metabolism | 4.02 | 4.33±0.36 | 1.04 | 2.09±0.31 | 3.76 | 3.91±0.58 |
| Gene4092 | Cysteine and methionine metabolism | 6.32 | 7.08±0.76 | 4.66 | 5.23±0.63 | -1.49 | -2.31±0.29 |
| Gene4973 | Metabolic pathways | 4.06 | 4.18±0.31 | 3.49 | 3.20±0.35 | 3.77 | 3.16±0.51 |
| Gene4057 | Cysteine and methionine metabolism | 2.98 | 2.54±0.23 | — | -0.25±0.20 | -2.09 | -2.54±0.43 |
| Gene1941 | Pyrimidine metabolism | 1.86 | 1.64±0.1 | 3.42 | 4.13±0.06 | 1.59 | 2.86±0.38 |

# Supplementary Figures


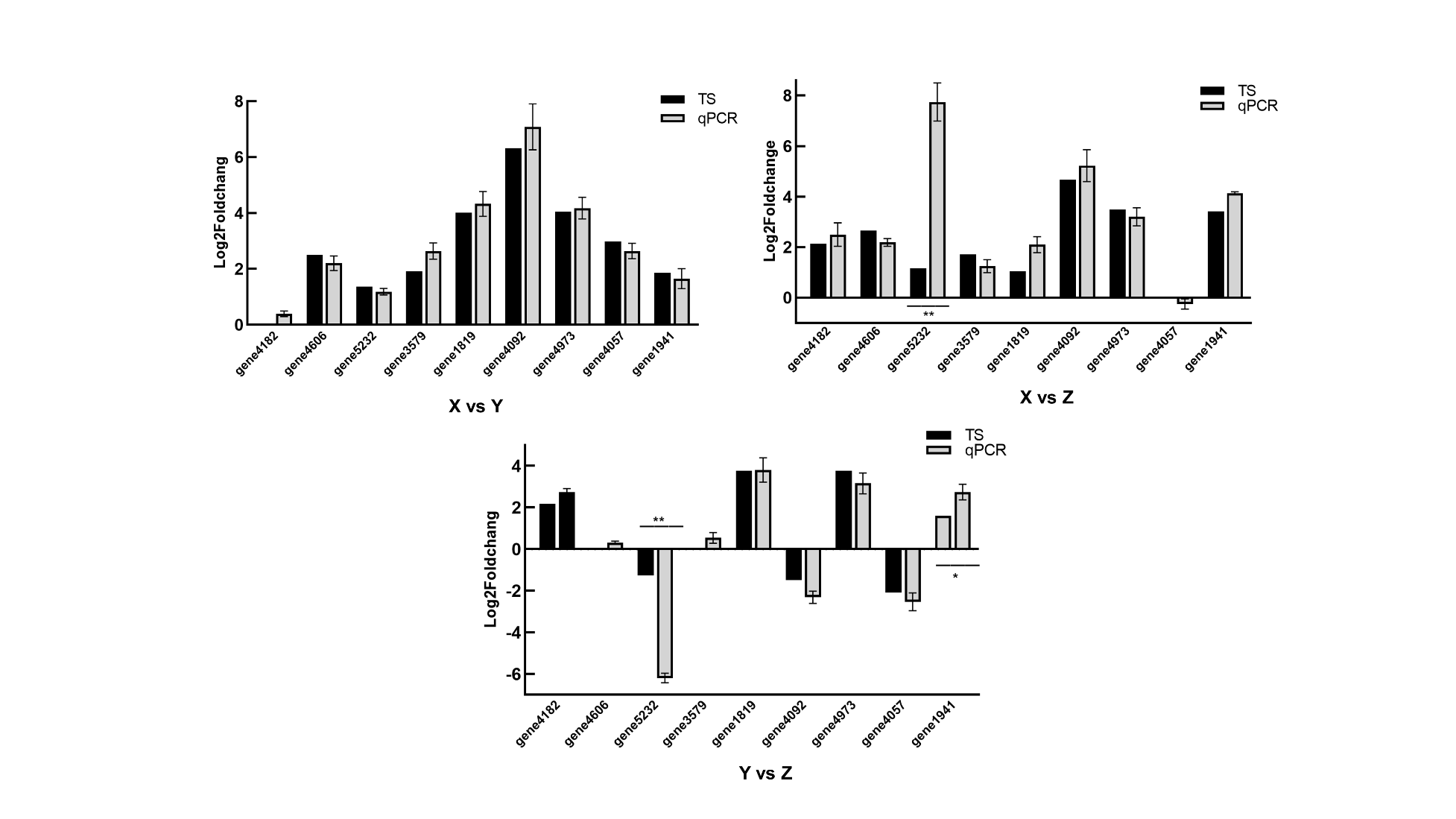


**Supplementary Figure 1.** Comparison of transcriptome data and RT-qPCR data in different groups. Note: TS is Transcription sequencing. X vs Y is TSB vs 10% TSB (RI in TSB vs RI in 10% TSB); X vs Z is RI in TSB vs RI and LM in 10% TSB; Y vs Z is RI vs RI with LM (RI in 10% TSB vs RI and LM in 10% TSB).
